# Quantitative proteomic analysis for novel biomarkers of buccal squamous cell carcinoma arising in background of oral submucous fibrosis

**DOI:** 10.1186/s12885-016-2650-1

**Published:** 2016-08-02

**Authors:** Wen Liu, Lijuan Zeng, Ning Li, Fei Wang, Canhua Jiang, Feng Guo, Xinqun Chen, Tong Su, Chunjiao Xu, Shanshan Zhang, Changyun Fang

**Affiliations:** 1 Department of Oral and Maxillofacial Surgery, Xiangya Hospital, Central South University, No. 88, Xiangya Road, Changsha, China; 2 Department of Oral Medicine, Xiangya Hospital, Central South University, No. 88, Xiangya Road, Changsha, China

**Keywords:** Oral submucous fibrosis, Buccal squamous cell carcinoma, Quantitative proteomic analysis, Annexin A4, Filamin-A

## Abstract

**Background:**

In South and Southeast Asian, the majority of buccal squamous cell carcinoma (BSCC) can arise from oral submucous fibrosis (OSF). BSCCs develop in OSF that are often not completely resected, causing local relapse. The aim of our study was to find candidate protein biomarkers to detect OSF and predict prognosis in BSCCs by quantitative proteomics approaches.

**Methods:**

We compared normal oral mucosa (NBM) and paired biopsies of BSCC and OSF by quantitative proteomics using isobaric tags for relative and absolute quantification (iTRAQ) to discover proteins with differential expression. Gene Ontology and KEGG networks were analyzed. The prognostic value of biomarkers was evaluated in 94 BSCCs accompanied with OSF. Significant associations were assessed by Kaplan-Meier survival and Cox-proportional hazards analysis.

**Results:**

In total 30 proteins were identified with significantly different expression (false discovery rate < 0.05) among three tissues. Two consistently upregulated proteins, ANXA4 and FLNA, were validated. The disease-free survival was negatively associated with the expression of ANXA4 (hazard ratio, 3.4; *P* = 0.000), FLNA (hazard ratio, 2.1; *P* = 0.000) and their combination (hazard ratio, 8.8; *P* = 0.002) in BSCCs.

**Conclusion:**

The present study indicates that iTRAQ quantitative proteomics analysis for tissues of BSCC and OSF is a reliable strategy. A significantly up-regulated ANXA4 and FLNA could be not only candidate biomarkers for BSCC prognosis but also potential targets for its therapy.

**Electronic supplementary material:**

The online version of this article (doi:10.1186/s12885-016-2650-1) contains supplementary material, which is available to authorized users.

## Background

Oral submucous fibrosis (OSF) is a chronic and insidious lesion of oral mucosa which demonstrates particularly prevalent in some South and Southeast Asian countries [1, 2]. Its histopathologic feature is characterized by the inflammatory reaction of juxta-epithelial region followed by excessive collagen deposition of the lamina propria and the underlying submucosal layer, with associated epithelial atrophy [3]. A major clinical symptom of OSF patient is trismus, a limited ability to open the mouth, which eventually impairs eating, speaking and dental care [4, 5]. Various epidemiological studies have found that the chewing of areca-nut is the main etiological factor for OSF. [6].

OSF is associated with raised risk for the oral squamous cell carcinoma (OSCC), especially buccal SCC (BSCC), because buccal mucosa is the most common region that is stimulated by chewing areca nut [7–9]. The frequency of OSF canceration has been reported to range from 3 % to 6 % [10]. The oral precancerous condition defined by WHO is that a generalized pathological state of the oral mucosa associated with a significantly increased risk of cancer, which accords well with OSF characteristics [8]. Meanwhile, OSF is currently a public health problem in many countries, especially in some countries of southeastern Asian [11].

The molecular mechanisms of OSF progression and oncogenesis remain unclear and may be considered complex events in the deregulated expression of multiple molecules [12]. High-throughput proteomics can perform analysis to know expression profiles for thousands of proteins and characterize the biologic behaviors of cell simultaneously, which can contribute to better understand the changes of multiple proteins related to the disease progression and identify diagnostic and prognostic biomarkers. Different proteomics studies have been successfully engaged in the biomarker discovery of oral cancer. However, it is still hard to discover unique biomarkers to predict which oral mucosal disease will progress to OSCC [13, 14].

In the present study, we analyzed normal buccal mucosa (NBM), OSF and BSCC by isobaric tags for relative and absolute quantification (iTRAQ) system with two-dimensional liquid chromatography-tandem mass spectrometry (2DLC-MS/MS) to find the biomarkers contributed to the diagnosis and prognosis of OSF and BSCC. iTRAQ can label total peptide, preserve the information of post-translational modification and make quantitative analysis of 4 tissue samples simultaneously with same experimental conditions [14, 15]. Two novel protein biomarkers identified in our study may be clinically useful for BSCC detection arising from OSF, and evaluate their prognosis values.

## Methods

### Experimental design and analytical strategy

Briefly, there were three consecutive phases in this study: first a discovery screen of quantitative proteomics based on iTRAQ was carried out to identify candidate biomarkers with the consistently deregulated expressing levels from NBM to OSF to BSCC, second a protein-level evaluation of promising biomarkers by western blotting and immunohistochemistry, and third a validation of the candidate biomarkers in clinical samples by a retrospective study. We received ethical approval from the Xiangya Hospital Human Research Ethics Committee. All patients included for both the biomarker discovery screen and the retrospective clinical validation study were diagnosed with a primary BSCC arising from OSF. Enrolled cases were scheduled for surgical treatment with informed consent. Meanwhile, all cases had the habit of areca-quid chewing, and no previous local treatments for oral mucosa. All histological evaluations and grading were done according to the WHO standard criteria.

### Patients and Tissue Samples

Paired biopsies of BSCC and OSF tissue were collected from BSCC patient accompanied with OSF lesion simultaneously. For every patient, BSCC sample was taken from the surgical cancer tissue, and matched OSF sample was from the contralateral buccal mucosa. In addition, unmatched NBM tissue was procured from healthy volunteer without the habit of betel-quid chewing. Each specimen was divided into three parts: one was for pathologic review to confirm the diagnosis, while the remaining two parts were immediately snap-frozen for quantitative proteomic and western blotting analysis respectively. If a paraffin specimen was confirmed by pathologists, it would be stored for immunohistochemical analysis. Eventually, 6 NBMs, 6 OSFs and 6 BSCCs were enrolled for proteomic and western blotting analysis. Clinical and histopathologic details of enrolled cases are listed in Table 1. Ninety-four formalin-fixed paraffin-embedded BSCC specimens, which were all removed from primary BSCC patients accompanied with OSF between November 2008 to August 2013, were drawn and reconfirmed for the retrospective clinical validation study. Age, TNM grade, UICC classification, OSF and BSCC histological grade, and survival time were recorded as the clinicopathological data (Additional file 1: Table S1). All enrolled cases had the habit of areca-quid chewing. All histological evaluations were done according to the WHO standard criteria.

**Table 1:** BSCC patients enrolled for iTRAQ quantitative proteomic analysis

Case	Age Range	BSCC Site	BSCC	BSCC	OSF
	(y)		T-stage	Differentiation	Differentiation
1	30–35	Left	T_3_N_1_M_0_	Well	Moderate
2	35–40	Left	T_1_N_0_M_0_	Well	Moderate
3	45–50	Right	T_3_N_0_M_0_	Well	Early
4	25–30	Left	T_2_N_0_M_0_	Well	Moderate
5	55–60	Right	T_2_N_1_M_0_	Well	Advanced
6	45–50	Left	T_3_N_2_M_0_	Well	Advanced

### Reagents and apparatus

iTRAQ™ Reagents Kit was bought from Applied Biosystems (San Jose, CA, USA). The acetonitrile, formic acid, acetone, trypsilin, and sodium citrate buffer were from Sigma-Aldrich (California, CA, USA). The Zorbax 300SB-C18 reversed-phase column (Microm, Auburn, CA, USA), the Polysulfoethyl column (The Nest Group, Southborough, MA, USA) and QSTAR XL System (Applied Biosystem, California, CA, USA) were for 2D LC-MS/MS. Sep-Pak Vac C18 cartridges was obtained from Millipore Corporation (Minneapolis, Minnesota, USA). The rabbit polyclonal antibodies were purchased from Abcam (London, UK).

### Candidate biomarkers discovery by quantitative proteomic analysis

#### Protein preparation and iTRAQ labeling

The protein samples were quantitated by the Bradford method. The iTRAQ labeling was carried on according to the protocol. Briefly, 200 μg proteins were precipitated at-20 °C for 60 min. Then they were resuspended in 20 μl dissolution buffer. After reduction and alkylation, the peptides were labeled with iTRAQ regents for 60 min. Three iTRAQ regents (115, 116 and 117) were used to label the peptides of OSF, BSCC and NBM respectively. Sequentially, the samples were mixed together, and desalted by Sep-Pak Vac C18 cartridges.

#### 2D LC-MS/MS analysis

The mixed labeling peptides were fractionated by strong cation exchange chromatography (SCX). The mixture was reconstituted with Buffer A (10 mM KH_2_PO_4_ in 25 % acetonitrile, PH 2.6). The mixed peptides were separated at a 500 μl/min with a gradient of 0-80 % Buffer B (Buffer B was Buffer A containing 350 mM KCl) in Buffer A for 1 h. The 215 nm and 280 nm absorbance was monitored and a total of 12 SCX fractions were got together. The fractions were dried and resuspended in 50 μl HPLC Buffer A (5 % acetonitrile, 0.1 % formic acid). Then they were loaded across the Zorbax 300 SB-C18 reversed-phase column and assessed on a QSTAR XL System with a 20 AD HPLC system. The elution flow rate was 0.3 μl/min with gradient 5 %–35 % HPLC Buffer B (98 % acetonitrile, 0.1 % formic acid) for 90 min. The scans were obtained with m/z ranges of 400–1800 for MS with up to three precursors selected from m/z 100–2000 for MS/MS.

#### Protein identification

The MS/MS data were searched from the International Swissprot using the Protein Pilot software 3.0 (Applied Biosystem, USA). The parameters were as follows: trypsilin as enzyme, methylmethanethiosulphonate of cysteines residues as modification. Then the Paragon Algorithm followed by the ProGroup Algorithm (Applied Biosystem, USA) were used to cancel redundant hits. Parent ion accuracy, fragment ion mass accuracy, tryptic cleavage specificity, and allowance for missed cleavages were provided by Protein Pilot. The benchmark for protein identification was unused Prot-Score >1.3 (95 %) as the threshold. The relative protein expression was based on the ratio of peptides ions (115:117 or 116:115). We used the fold change ratio ≤ 0.5 or ≥2 to designate differentially expressed proteins (*P* < 0.05).

#### Bioinformatic analysis

Pathway analysis was performed by the Kyoto Encyclopedia of Genes and Genomes (KEGG) database. Gene Ontology (GO) database was used to facilitate the biological interpretation of the identified protein in these studies. The differentially expressed proteins of GO were divided into 3 categories as follows: biological process (BP), molecular function (MF) and cellular component (CC).

### Validation Studies

#### Western blotting

30 μg protein was firstly separated with 12 % SDS-PAGE, then transferred on the polyvinylidene fluoride (PVDF) membrane. After blocked, filter was incubated by the primary antibody. The secondary antibody (Santa Cruz Biotechnology, California, CA) was applied onto the filter at 1:2,000 dilutions. Samples were probed with antiβ-actin antibody (BD Biosciences, San Jose, CA) as an internal control. We used ECL system (Amersham, Buckinghamshire, UK) to visualize bands, and the Bandscan software (Glyko, Novato, CA) for the analysis of signal intensity.

#### Immunohistochemical evaluation

Briefly, serial 3 μm thick sections of tissue sample were mounted on silanized slides. After blocked by 3 % hydrogen peroxide, sections were incubated by primary antibodies, then by the biotinylated IgG (Santa Cruz Biotechnology, CA) for 30 min. Antigen–antibody complexes were dealed with diaminobentzidine (DAB). Then slides were counterstained by Mayer’s Hematoxylin. The immunoreactivity of candidate biomarkers were assessed by counting the number of positive cells. We considered that ≥10 % positive cells were graded as immunopositive. For every sample, the result of immunoreactive staining was evaluated by two observers blinded for the data.

### Clinical and prognostic validation in a retrospective case study

#### Cohort for the retrospective study

Ninety–four primary BSCCs accompanied with OSFs were immunohistochemically stained for biomarker candidates.

#### Follow-up study

All patients undergoing surgery were followed up. The time to death or recurrence was recorded in detail periodically. Disease free survival time was recorded from the time of histological diagnosis to the time of the last follow-up. If a patient died or was found recurrent, survival time was censored at that time. Overall survival can not be regarded as a separate parameter, because among the patients lost to follow up, the death number could not be ascertained. Only disease free survival of the patients was recorded.

### Statistical Analysis

Statistical analysis of western blotting data was dealed with Student’s *t* test. The relationship between the expression of proteins and clinicopathological parameters was evaluated by Chi-Square or Fisher’s exact test. Follow-up studies were evaluated by Kaplan–Meier and Cox’s Proportional Hazards test. *P* < 0.05 was regarded as significant. All statistical analysis was performed by SPSS 19.0.1 software.

### Ethics statement

This study has been approved by the Ethics Board of Xiangya Hospital, which was also in accordance with the 1975 Helsinki Declaration. All patients had written the informed consent. Human samples were performed anonymously.

## Results

### Biomarker discovery screen

A total of 1998 proteins were identified from 14237 peptides among three tissues, based on the Unused ProtScore >1.3 (95 %) with at least one peptide above the 95 % confidence. 71.7 % proteins were with at least two peptides. And 56.2 % proteins were identified with three or more (Additional file 2: Table S2). Compared NBM 117 labeled, 90 proteins were up-regulated and 46 were down-regulated significantly in OSF 115 labeled. Meanwhile, between BSCC 116 labeled and OSF 115 labeled, 91 differential proteins were obtained, which contained 51 up-regulated and 40 down-regulated proteins in BSCC. Most importantly, in total of 30 proteins were identified with significantly different expression among three tissue types (Table 2). Among them, 2 candidate proteins (Annexin A4, ANXA4; Filamin-A, FLNA) were consistently upregulated, and one protein (Fibrinogen alpha chain precursor, FGA) was consistently down-regulated from NBM to OSF to BSCC.

**Table 2:** Total 30 differentially expressed proteins among three tissue types

Protein Symbol	Accession	Fold Change	
		BSCC/OSF (116:115)	OSF/NBM (115:117)
**ANXA4** ^*^	**IPI00872780.1**	**4.8305881**	**2.051162243**
MFAP4	IPI00793751.1	0.033419501	5.7543993
GATM	IPI00792191.1	3.250873089	0.343557954
CES1	IPI00607801.2	11.16862965	0.108642563
PSME1	IPI00479722.2	3.837071896	0.296483129
KRT19	IPI00479145.2	0.197696999	18.03017807
HIST1H4I	IPI00453473.6	0.04786301	2.582260132
VIM	IPI00418471.6	0.296483099	2.558585882
**FLNA** ^*^	**IPI00333541.6**	**3.83707315**	**2.128139019**
KRT7	IPI00306959.10	0.246603906	4.285485268
COL1A2	IPI00304962.3	0.343558013	2.167704105
COL1A1	IPI00297646.4	0.27289781	2.884031534
GPD1	IPI00295777.6	0.2511885881	4.055085182
LTB4DH	IPI00292657.3	3.630779982	0.405508548
COL6A1	IPI00291136.4	0.366437614	2.83139205
MPO	IPI00236556.1	0.205116197	2.466039419
GOT1	IPI00219029.3	0.465586096	8.709635735
ADH4	IPI00218899.5	0.2679167986	5.649369717
GSTM1	IPI00218831.4	0.2051162004	9.549925804
CALM1	IPI00075248.11	0.35318321	13.55189419
CTSG	IPI00028064.1	0.251188606	5.105050087
HSP90B1	IPI00027230.3	2.83139205	0.310455948
PDIA3	IPI00025252.1	3.40408206	0.237684026
TF	IPI00022463.1	2.754229069	0.322106868
APCS	IPI00022391.1	0.110662401	4.830587864
**FGA** ^*^	**IPI00021885.1**	**0.387257606**	**0.432513833**
P4HB	IPI00010796.1	3.28095293	0.280543357
PDIA4	IPI00009904.1	3.908409119	0.187068209
EPHX1	IPI00009896.1	3.80189395	0.157036275
HSPA5	IPI00003362.2	2.77971292	0.23120648

^*^The proteins written with bold words were the same differentially expressed proteins among three tissue types (from BSCC to OSF to NBM)

### KEGG pathway analysis

**Fig. 1:**
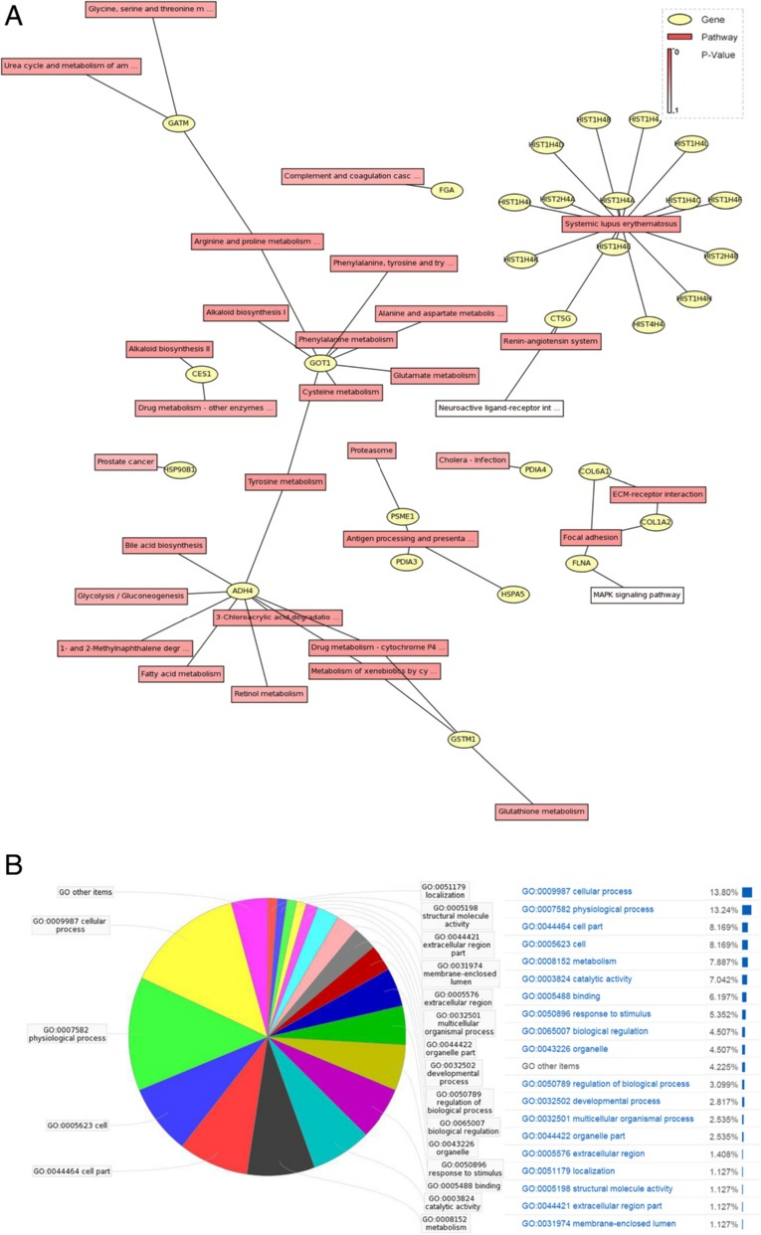
Bioinformatic analysis of differentially expressed proteins. **a** KEGG pathway analysis of the network relationships between proteins and related pathways. Red boxes indicate differentially expressed proteins, and yellow circles indicate the related pathways. The depth of red color shows the *p*-value which indicates the enrichment of proteins in the pathway. **b** pie graph of GO mapping for differential expression proteins. Cellular process GO term accounted for the top GO term, then the physiological process and cell part

Thirty–two signaling pathways among three tissue types were identified using KEGG database (Fig. 1a). The differentially expressed protein clusters could be assigned into numerous subcategories including the systemic lupus erythematosus, antigen processing and presentation, arginine and proline metabolism, focal adhesion, tyrosine metabolism, and so on. There were cross-talks among these pathways, as one protein might participate in several signaling pathways. Alcohol dehydrogenase 4 (ADH4) was involved in the most pathways (9 pathways) and Systemic lupus erythematosus pathway accounted for the most differentially expressed proteins (15 proteins) (Additional file 3: Table S3).

### GO analysis

These differentially expressed proteins were grouped into 72 (45.28 %) GO terms based on BP GO terms. The most enriched BP GO terms included cell redox homeostasis, interspecies interaction between organisms and oxidation reduction. There were 52 (32.7 %) GO terms identified by MF classification, and 35 (22.01 %) GO terms identified by CC classification. The top component for MF were protein binding, which consisted of 7 proteins. While the top component for CC were cytoplasm, which also consisted of 7 proteins (Additional file 4: Table S4).

On the other hand, as shown in Fig. 1b, cellular process (13.80 %) GO term which belongs to BP classification accounted for the top GO term, then the physiological process (13.24 %) and cell part (8.169 %).

### Initial evaluation of candidate biomarkers

ANXA4 and FLNA were selected as the candidate biomarkers for BSCC arising OSF lesion because the two showed consistently upregulated from NBM to OSF to BSCC.

### Western blotting

**Fig. 2:**
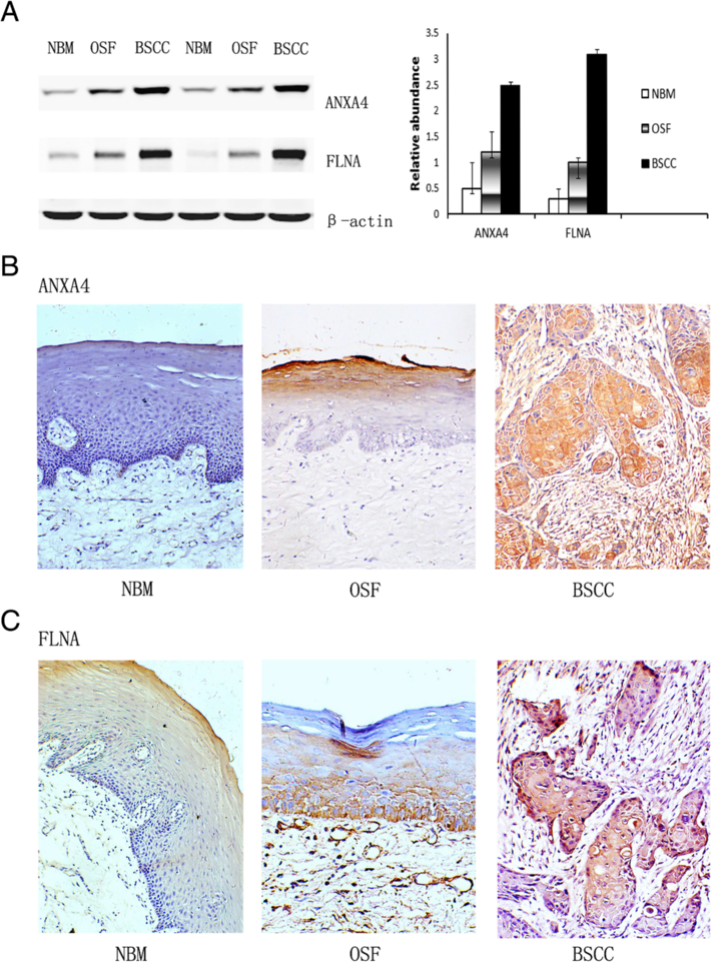
Initial validation of two candidate biomarkers. **a** Western blot of ANXA4 and FLNA in the samples of NBM and paired BSCC and OSF, as well as their corresponding quantifications. **b** representative immunohistochemical staining for ANXA4. Negative expression of ANXA4 in NBM, brown cytoplasm staining limited to the spinous epithelial layer of OSF, and intensively staining of the cytoplasm in BSCC cell nest. **c** representative immunohistochemical staining for FLNA. Weak expression in NBM samples, brown cytoplasm staining limited to the lower spinous epithelial layer and basal cell layer of OSF, and intensively staining of the cytoplasm in BSCC cell

Staining intensities of ANXA4 and FLNA in BSCC were all significantly higher than OSF and NBM with a consecutively upregulated trend from NBM to OSF to BSCC (*P* = 0.002 and 0.001, respectively). Representative results were presented in Fig. 2a.

### Immunohistochemistry evaluation

In Fig. 2b, no detectable expression of ANXA4 was found in NBM, while OSF exhibited brown cytoplasm staining mainly limited to the spinous epithelial layer, and sometimes keratinocyte layer together. While in the BSCC, ANXA4 protein showed intensively staining of the cytoplasm in cancer cell. The positive expression of ANXA4 in BSCC was significantly higher than OSF (*P* = 0.008), while positive ANXA4 of OSF was significantly higher than NBM (*P* < 0.0001).

In Fig. 2c, very weak expression of FLNA was shown in NBM. However, OSF exhibited brown cytoplasm staining mainly limited to the lower spinous epithelial layer and basal cell layer. While in the BSCC, FLNA protein showed intensively staining of the cytoplasm in cancer cell. The positive expression of FLNA in BSCC was significantly higher than OSF (*P* = 0.004), while positive FLNA expression in OSF tissues was significantly higher than NBM tissues (*P* = 0.01).

### Correlation of candidate biomarkers with clinicopathological parameters

As shown in Table 3, positive ANXA4 and FLNA were significantly related to T stage (*P* = 0.017 and *P* = 0.042, respectively). Positive ANXA4 showed a forward relationship with N stage (*P* = 0.001), while positive FLNA showed an inverse trend with N stage (*P* = 0.017). Meanwhile, there was a statistically significant relationship between positive ANXA4 and tumor stage (*P* = 0.004), while no association was found in other parameters.

**Table 3:** Correlation with clinicopathologic characteristics of the patients and immunostaining of ANXA4 and FLNA (*n* = 94)

	Cases	ANXA4 (+)(%)	*p* value	FLNA (+)(%)	*p* value
Gender					
Male	83	59(71.1)	0.455	57(68.7)	0.425
Female	11	9(81.8)		5(45.5)	
Age					
≤40 yr	42	32(76.2)	0.453	28(66.7)	0.896
>40 yr	52	36(69.2)		34(65.4)	
T stage					
T1	8	3(37.5)	0.017^*^	2(25)	0.042^*^
T2	44	29(65.9)		28(63.6)	
T3	36	30(83.3)		27(75)	
T4	6	6(100)		5(83.3)	
N Stage					
N0	48	27(56.3)	0.001^*^	38(79.2)	0.017^*^
N1	43	38(88.4)		23(53.5)	
N2	3	3(100)		1(33.3)	
UICC Stage					
I	7	2(28.6)	0.004^*^	4(57.1)	0.696
II	31	19(61.3)		19(61.3)	
III	50	41(82)		34(68)	
IV	6	6(100)		5(83.3)	
Diff.					
Well	80	59(73.8)	0.652	55(68.8)	0.180
Moderate	10	6(60)		6(60)	
Poor	4	3(75)		1(25)	
OSF Stage					
Early	27	22(81.5)	0.294	16(59.3)	0.278
Moderated	46	30(65.2)		34(73.9)	
Advanced	21	16(76.2)		12(57.1)	

^* P < 0.05^

### Association of candidate biomarkers with patient prognosis

**Fig. 3:**
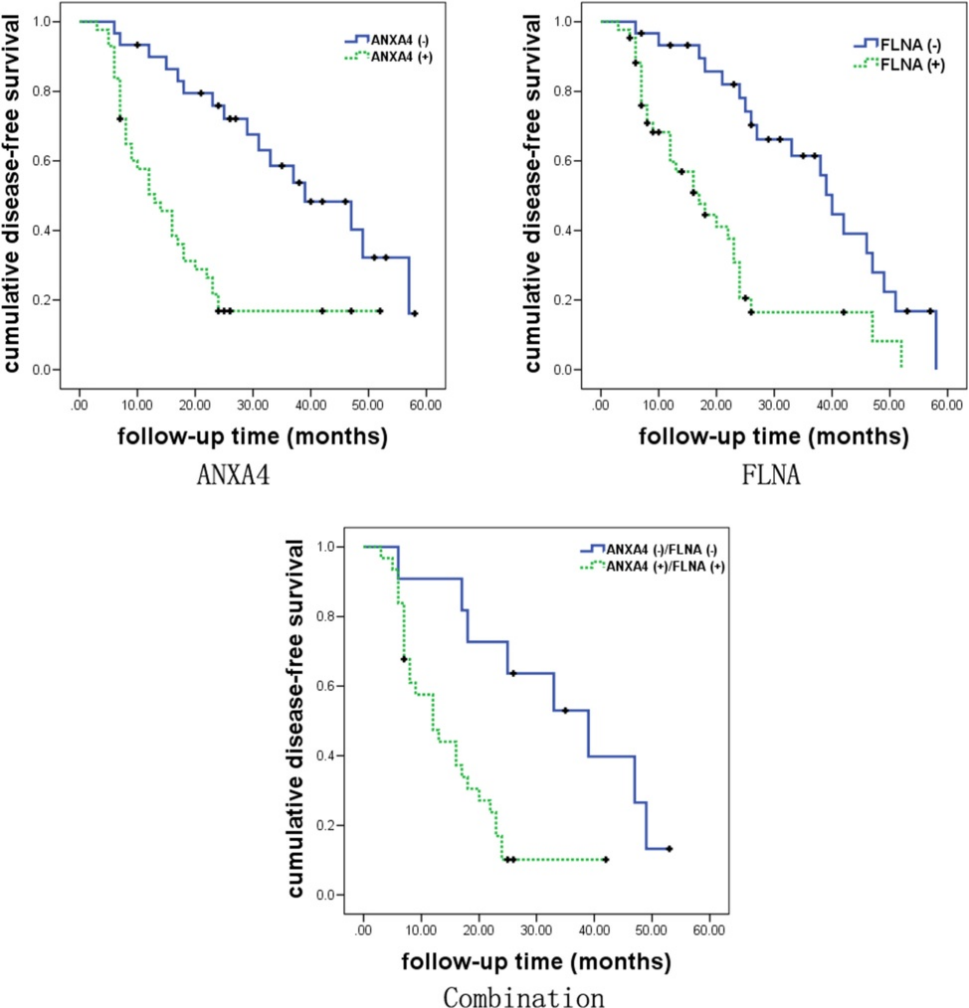
Kaplan-Meier curves of local disease free survival of BSCC patients accompanied with OSF in relation to ANXA4 staining, FLNA staining, and the combination of both

Seventy–three of 94 BSCC patients could be followed up. Patients were monitored for a period of median 22 months and a maximum of 58 months. Kaplan-Meier curves revealed that the disease-free survival was associated significantly with the negative expression of ANXA4 and FLNA (*P* = 0.000 and *P* = 0.000, respectively) in BSCCs in Fig. 3. Hazard ratios calculated by univariate Cox regression analysis, were 3.4 (95 % confidence interval, 2.2–7.5; *P* = 0.004) for ANXA4 and 2.1 (95 % confidence interval, 1.7–5.5; *P* = 0.0036) for FLNA. ANXA4 and FLNA immunostaining data were combined to form one BSCC group with positive ANXA4 and FLNA expression, and another group with negative ANXA4 and FLNA. This classification showed an association between patients with negative ANXA4 and FLNA and disease-free survival (*P* = 0.002) and has a superior prognostic power with a hazard ratio of 8.8 (95 % confidence interval, 3.0–32.6; *P* = 0.005).

## Discussion

Some previous studies have identified a large number of differentially expressed biomarkers at the mRNA level between normal oral mucosa and OSCC or OSF tissues respectively [16–19]. Meanwhile lots of protein biomarkers between normal oral mucosa and OSCC have also been found for long time. However, few studies focused the differentially expression of protein biomarkers between NBM and OSF. The present study is the first comprehensive research on proteins with differential expression among NBM, OSF and BSCC arising from OSF by using the iTRAQ shot-gun proteomic approach [20]. In this present study, we used whole tissue rather than microdissected tissue cells for our proteomics analysis. We think that whole tissue could have the ability of reflecting the tumor microenvironment accurately, which is believed to determine whether cancer can spread through epithelial-mesenchymal interactions (EMT) [21]. However, the main limitation for whole tissue in proteomics analysis is the cell heterogeneity of different tissues.

By iTRAQ proteomic approach, we identified in total 30 unique proteins from NBM to OSF to BSCC. Among the deregulated proteins, some were previously reported to be correlated with the pathogenesis of OSF, such as KRT19 [16], COL1A2 [22], GSTM1 [23], VIM; [24] some were not yet observed in OSF but within OSCC, for instance PSME1 [25], FLNA [26], GOT1 [27], GSTM1; [28] and some were not reported in any study on both OSF and OSCC. In addition, a large number of proteins identified in the previous reports were not found in our present study. The discordance between them may be explained partially by the limited dynamic range of iTRAQ [15]. Moreover, the difference of races and region distributions, the different processed methods of areca nut, as well as the different procedure of tissue collection and management may contribute to the distinction among various laboratories.

The location, function and regulation of the differentially expressed proteins can be better and easier to understand by bioinformatics analysis. The results of bioinformatic analysis showed that most consistently expressed proteins were randomly regulated proteins during OSF pathogenesis and carcinogenesis, because most of them were found in the discrete interaction networks. The top 5 GO components showed that the differentially expressed proteins in the present study were located mainly in cytoplasm with the protein binding function, which contained cell redox homeostasis, interaction between organisms, oxidation reduction and tissue regeneration. The top regulation network in the study, systemic lupus erythematosus pathway, indicated that immunological reaction might be the most important factor during the pathogenesis and carcinogenesis of OSF lesion, which is in agreement with the conclusions of our previous study and other research groups [16, 29–31].

Notable proteins in our present study were three consistently deregulated proteins from NBM to OSF to OSCC, which were related to the mechanisms of the progression of OSCC arising from OSF. Two consistently upregulated proteins, ANXA4 and FLNA, were selected as the candidate biomarkers because we considered that the progress of OSF pathogenesis and carcinogenesis could be blocked effectively through interfering their upregulated expression. They would be promising targets for molecular therapy of OSF and OSCC.

The annexins, a multigene family of calcium-dependent phospholipid-binding proteins, have some special functions include the aggregation of vesicles and regulation of ion channels as well as roles in the regulation of cell cycle, cell signal and cell differentiation [32]. Meanwhile, annexins have been found in the processes of several disease, involving in inflammation and several neoplasia [33]. Of all annexins, ANXA4 was related to the loss of cell adhesion, and play important roles in apoptosis, carcinogenesis, chemoresistance, migration and invasion of cancer cells [34]. It binds phospholipids through the Ca-dependent manner and is located in the nucleus, cytoplasm, or membrane of cell [35]. ANXA4 was overexpressed in various primary clinical epithelial tumors, such as renal cancer [34], ovary cancer [35], gastric cancer [36], colorectal cancer [37], breast cancer [38], laryngeal carcinoma [38], pancreatic cancer [38, 39]. Its overexpression could enhanced significantly with the tumor stage and poorer prognosis [39], and be related to promote cell migration in a model tumor system [37]. These results are correlates with our observation in the present study that increased ANXA4 expression is associated with BSCC stage and poor prognosis. ANXA4 can form protein kinase C complexes. Moreover, it is found that at least 10 isoforms of protein kinase C have roles in the progression of cancers, including OSCC [40]. It could be found association with protein kinase C that ANXA4 has a vital effect on the BSCC pathogenesis. All these findings indicate that ANXA4 might have a vital role in the BSCC progression and migration. Meanwhile, ANXA4 expression was first identified in OSF tissues, which further proved the potential carcinogenic capacity of OSF.

FLNA is a type of actin filament cross-linking protein that participates in cytoskeletal rearrangement [41]. By its scaffolding function, FLNA can interact with more than 90 functionally diverse binding partners to regulate cellular functions and processes [42, 43]. The FLNA-deficient cells can not polarize and move because of their unstable surfaces which can continuously expand and contract circumferential blebs [44]. The orthogonal networks of FLNA have the active and reversible organizational properties, which can protect cell from various shear stresses [45]. In the present study, we firstly found that FLNA was positively expressed in OSF. Obviously, for oral mucosa cells in OSF patients, persistently mechanical shear stress caused by areca-nut chewing could be the key reason for the upregulated FLNA as a protective reaction of oral mucosa. Mis-regulation of FLNA plays a critical role in DNA double strand breaks response for the initiation of tumorigenesis [46]. Meanwhile, because of its ability to control cell mobility, cell-ECM interactions, cell signaling, and DNA damage response, FLNA could be regarded as a novel biomarker for the diagnosis and outcome prediction of cancer. Meanwhile, it has been reported that there was the correlation of increased FLNA expression in different stages of various cancer types and patient outcomes, such as colorectal cancer [47], pancreatic cancer [48], gliomas [49], prostate cancer [50] and salivary gland adenoid cystic carcinoma [51]. In the present study, we employed quantitative proteomic analysis to assess the FLNA expression and localization. Our data also illustrated that the expression of FLNA was increased in BSCC, and a poor survival index for patients with BSCC have high FLNA levels. So it is conceivable that the FLNA level in BSCC can be developed as a promising biomarker for the outcome prediction of BSCC.

## Conclusion

Taken together, our proteome analysis has revealed a number of potential biomarkers among NBM, OSF and BSCC. Meanwhile, of these, ANXA4 and FLNA seem to have large prognostic value for patient survival, which may represent OSF and BSCC biomarkers and potential targets for therapeutical intervention. To our knowledge, although ANXA4 and FLNA has been reported on the carcinogenic roles of some tumors, no studies has been published on their expression in BSCC arising from OSF. However, more large-scale, prospective multicenter trials should be carried out to further elucidate their value in the clinic, and the roles of two biomarkers in BSCC development and invasion are in need of further study.

## Abbreviations

2-DE, two-dimensional gel electrophoresis; 2DLC-MS/MS, two-dimensional liquid chromatography-tandem mass spectrometry; ADH4, Alcohol dehydrogenase 4; ANXA4, Annexin A4; BP, biological process; BSCC, buccal squamous cell carcinoma; CC, cellular component; DAB, diaminobentzidine; EMT, epithelial-mesenchymal interactions; FGA, Fibrinogen alpha chain precursor; FLNA, Filamin-A; GO, Gene Ontology; ICAT, isotop-encoded affinity tags; iTRAQ, isobaric tags for relative and absolute quantification; KEGG, Kyoto Encyclopedia of Genes and Genomes; MF, molecular function; NBM, normal oral mucosa; OSCC, oral squamous cell carcinoma; OSF, oral submucous fibrosis; PVDF, polyvinylidene fluoride; SCX, strong cation exchange chromatography; SILAC, stable isotope labeling by amino acids in cell culture

## Additional files

Additional file 1: Table S1. Age, TNM grade, UICC classification, BSCC histological grade, OSF histological grade, survival status and time, and the IHC expression of ANXA4 and FLNA were recorded as the clinicopathological data of 94 cases. Additional file 2: Table S2. There are four excel files in the supplement table S2. No. 1 is “total proteins”, which presents all identified proteins among NBM, OSF and BSCC. No.2 is “DP-(115–117)”, which presents the differential proteins identified between OSF (115) and NBM (117). Red proteins mean the upregulated differential proteins in OSF with the change fold (115:117) > 2, while the blue proteins mean the downregulated proteins in OSF with the change fold (115:117) < 0.5. No.3 is “DP-(116–115)”, which presents the differential proteins identified between BSCC (116) and OSF (115). Red and blue proteins mean the up–or down–regulated proteins respectively in BSCC. No.4 is “DP-(116–115–117)”, which presents the differential proteins identified among BSCC (116), OSF (115) and NBM (117). Red proteins mean consistently upregulated ones, and blue one was consistently down-regulated from NBM to OSF to BSCC. (XLS 725 kb) Additional file 3: Table S3. KEGG pathway analysis was done for 30 differential proteins from BSCC to OSF to NBM. There are 2 excel files in the supplement table S3. No. 1 is “pathway indexe by Pathway_kegg”, which presents 32 pathways in total 30 proteins and the pathway of Systemic lupus erythematosus contains the most proteins. No. 2 is “pathway indexe by Symbol”, which presents ADH4 contains the most pathways. (XLS 22 kb) Additional file 4: Table S4. GO analysis was done for 30 differential proteins from BSCC to OSF to NBM. There are 3 excel files in the supplement table S4. No. 1 is “go indexe by GO_molecular_ function”, which presents that in the molecular function protein binding contains the most proteins. No. 2 is “go indexe by GO_biological_process”, which presents that in the biological process cell redox homeostasis contains the most proteins. No. 3 is “go indexe by GO_cell_component”, which presents that in the cell component cytoplasm contains the most proteins. (XLSX 20 kb)
